# Relief of Suspected IgA Vasculitis-Associated Enterocolitis Manifested as Severe Diarrhea and Hypoalbuminemia Treated With Glucocorticoid Therapy and Administration of Fibrogammin® (Purified Coagulation Factor XIII Concentrate): A Case Report

**DOI:** 10.7759/cureus.89316

**Published:** 2025-08-04

**Authors:** Takashi Aikawa, Takashi Negishi, Tomohiro Iida

**Affiliations:** 1 Internal Medicine, Kiryu Kosei General Hospital, Kiryu, JPN

**Keywords:** coagulation factor xiii activity, enterocolitis, fibrogammin, immunoglobulin a vasculitis, intractable diarrhea

## Abstract

A 62-year-old female was admitted to our hospital with abdominal pain, diarrhea, and bloody stool. She suffered from severe diarrhea 30 times per day and consequently got hypoalbuminemia and hyponatremia. Esophagogastroduodenoscopy and total colonoscopy showed diffuse erosion of the duodenum, terminal ileum, and colorectum. Her endoscopic findings were similar to the former case reports of immunoglobulin A (IgA) vasculitis-associated enterocolitis. Further investigation of biopsy demonstrated that IgA-positive lymphocytes invaded the perivascular of the submucosal layer. However, we did not find IgA deposition in the capillary wall, and no granuloma, basal plasma cytosis, or crypt abscess was detected. Although a definite diagnosis could not be established, we considered the possibility of refractory IgA vasculitis-associated enterocolitis and initiated glucocorticoid therapy and administration of Fibrogammin® (purified coagulation factor XIII concentrate). The patient’s diarrhea improved within one and a half months after admission.

## Introduction

Immunoglobulin A vasculitis (IgAV), formerly called Henoch-Schoenlein purpura, is the most common systemic vasculitis in children, characterized by palpable purpura without thrombocytopenia, and may manifest as coagulopathy, arthralgia, abdominal pain, and kidney disease [1]. Gastrointestinal symptoms, such as periumbilical or epigastric pain, occur in up to 85% of IgAV patients, and this involvement can manifest as severe problems such as intussusception, obstruction, and perforation [2]. Although gastrointestinal symptoms typically develop within one week of the appearance of the rash, some case reports described that gastrointestinal symptoms were manifested without the appearance of cutaneous purpura at any time [3-5]. In such cases, IgAV could be difficult to diagnose.

We herein report a case in which suspected IgAV was relieved by the glucocorticoid therapy and the administration of Fibrogammin® (purified coagulation factor XIII concentrate).

## Case presentation

Clinical course

A 62-year-old female had a malaise without any symptoms of infection. Two days later, she suffered from abdominal pain, diarrhea, and bloody stools. Another two days later, she was admitted to our hospital with complaint of severe diarrhea occurring every 10 minutes. She had no relevant past medical history, surgical history, or allergy. She was also not on any medication. She had not consumed raw fish or undercooked meat within the preceding two weeks and did not own any pets.

**Figure 1:**
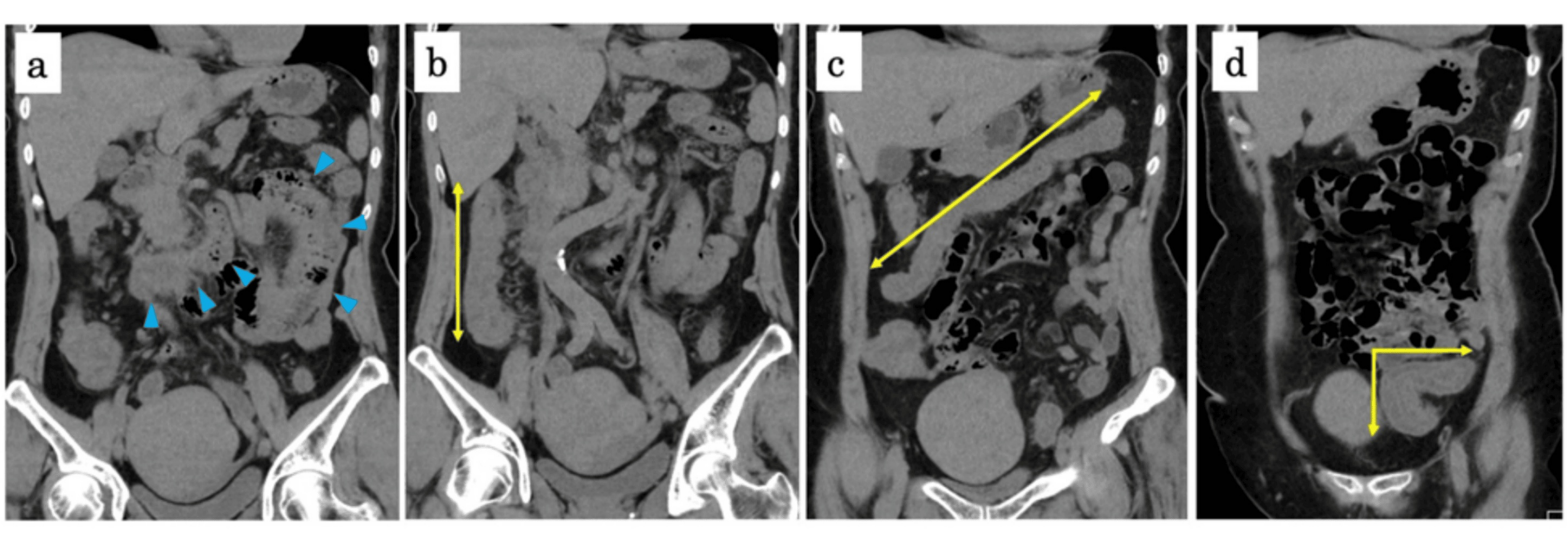
CT on admission Abdominal CT showing thickened wall of the transverse part of duodenum and proximal jejunum (a, blue arrowheads), ascending colon (b, yellow arrow), transverse colon (c, yellow arrow), and sigmoid colon (d, yellow arrow).

On the presentation, the patient’s vital signs were as follows: body (axillary) temperature of 36.1°C, pulse rate of 95 beats per minute, blood pressure of 192/128 mmHg, and oxygen saturation of 98 % on ambient air. She had cyclic abdominal pain and tenesmus. She did not manifest skin rash or arthralgia. The laboratory evaluation showed elevated C-reactive protein (CRP) (Table 1). Abdominal computed tomography (CT) showed moderate thickening of the intestinal wall from the cecum to the rectum, along with moderate enlargement of the perintestinal lymph nodes (Figure 1). The ER doctor suspected bacterial enterocolitis, and she was admitted to our hospital.

**Table 1:** Laboratory findings after admission The laboratory evaluation on admission showed a high CRP level without electrolyte imbalance or renal failure. As the patient's diarrhea lasted for three weeks, she had low levels of serum albumin, sodium, and chloride, along with abnormal blood coagulation. After discharge, the laboratory results returned back to normal values. *LRG is the serum biomarker to evaluate the activity of Crohn's disease or ulcerative colitis. At the beginning of our treatment, our differential diagnoses included not only autoimmune vasculitis but also Crohn's disease. CRP, C-reactive protein; eGFR, estimated glomerular filtration rate; FDP-D-dimer, fibrinogen degradation products fragment D-dimer; LRG, leucine-rich alpha 2 glycoprotein; PT-INR, prothrombin time-international normalized ratio

Day after admission	Day 1	Day 22	Day 62
Complete blood cell			
White blood cell (/μL)	8,400	5,500	6,500
Neutrophil (/μL)	-	3,800	4,000
Lymphocyte (/μL)	-	1,300	2,000
Eosinophil (/μL)	-	0.0	0.0
Hemoglobin (g/dL)	15.3	12.4	12.2
Platelet count (x10^4^/μL)	25.8	13.7	26.7
Biochemistry			
Total protein (g/dL)	6.9	4.2	4.9
Albumin (g/dL)	4.0	1.9	3.2
Lactate dehydrogenase (IU/L)	182	252	185
Creatine phosphokinase (U/L)	204	17	14
Total bilirubin (g/dL)	0.7	0.5	0.6
Aspartate aminotransferase (IU/L)	20	50	15
Alanine aminotransferase (IU/L)	13	96	34
Alkaline phosphatase (IU/L)	78	160	90
γ-glutamyl transpeptidase (IU/L)	21	320	59
Uric acid (mg/dL)	-	2.1	-
Blood urea nitrogen (mg/dL)	15	8	15
Creatinine (mg/dL)	0.59	0.47	0.55
eGFR (mL/min/1.73^2^)	78.1	100.2	84.0
Sodium (mEq/L)	137	126	142
Chloride (mEq/L)	100	95	104
Potassium (mEq/L)	3.9	4.4	3.8
CRP (mg/dL)	3.98	1.69	0.04
Ferritin (ng/dL)	-	1,205.4	480.2
sIL-2R (U/mL)	-	3,370	-
LRG (μg/mL)*	-	44.7	17.4
Coagulation test			
PT-INR	1.1	1.0	0.9
FDP-D-dimer (μg/mL)	-	3.73	0.96
coagulation factor XIII activity (%)	-	41	92
Urine examination			
Microalbuminuria (mg/L)	-	<1.0	-
Creatinine (mg/dL)	-	15.37	-
Sodium (mEq/L)	-	<10	-
Potassium (mEq/L)	-	6.6	-
Urine urea nitrogen (mg/dL)	-	132	-
β2-microglobulin (mg/L)	-	2,075	-

Subsequently, the patient was put on an intravenous drip without meal for bowel rest. In accordance with principles of moderate bacterial enterocolitis, she did not receive antibiotic therapy or antidiarrheal agent. After admission, she continued to suffer from severe diarrhea, with around 30 episodes per day. The laboratory findings showed low levels of serum albumin, sodium, and chloride (Table 1). The culture of feces that had been taken on admission did not have infectious bacterium.

Endoscopic findings

**Figure 2:**
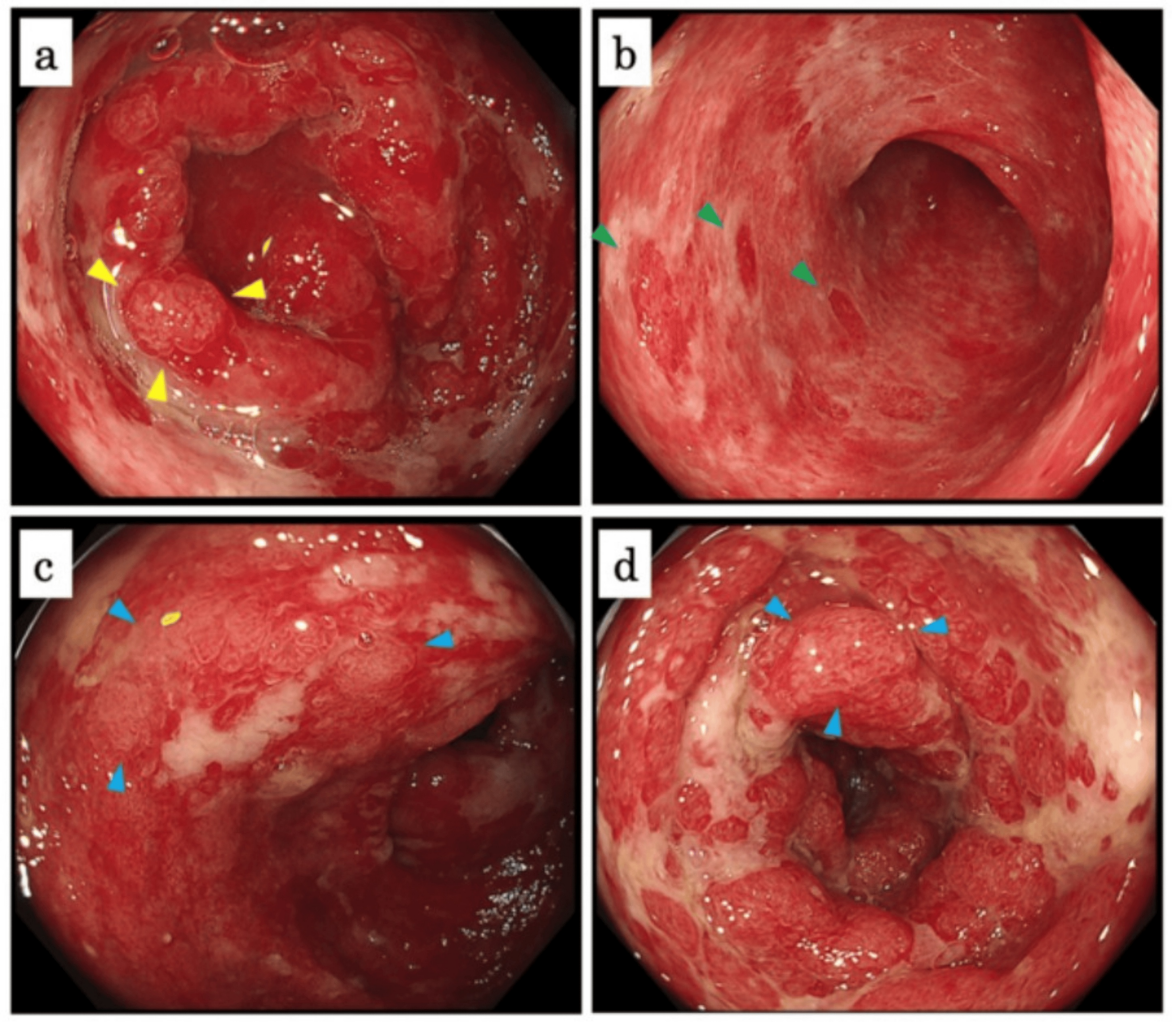
Total colonoscopy on day 14 after admission (a) The terminal ileum. (b-d) The colon. There was loss of the villi in the terminal ileum. There were small islands of the remaining villi in the terminal ileum (a, yellow arrowheads). There were a couple of round redness similar to hematoma-like protrusion in the colon (b, green arrowheads). In the colon, there were small islands, which looked alike shapes in the terminal ileum (c, d, blue arrowheads).

On day 14 after admission, we examined her with total colonoscopy (TCS), which showed mucosal erythema, edema, and multiple irregular ulcers with white slough. There was also loss of the villi in the terminal ileum, and there were a couple of round redness similar to hematoma-like protrusion in the colon (Figure 2). Endoscopic findings suggested the differential diagnosis of ulcerative colitis or autoimmune vasculitis instead of bacterial enterocolitis. Although we examined autoantibodies after TCS, antinuclear and anti-neutrophil cytoplasmic antibodies were negative (Table 2).

**Table 2:** Laboratory findings of serology and immunochemistry The laboratory findings did not imply any autoimmune disease including ANCA-associated vasculitis. Serology and bacterial culture did not show any infectious disease. HB, hepatitis B; HCV, hepatitis C virus; Ig, immunoglobulin; MPO-ANCA, myeloperoxidase-anti neutrophil cytoplasmic antibody; PR3-ANCA, proteinase 3-anti neutrophil cytoplasmic antibody; SARS-CoV RT-PCR,  severe acute respiratory syndrome coronavirus-2 reverse transcriptase-polymerase chain reaction

Laboratory findings	
Serology	
HBs antigen	Negative
HBs antibody	Negative
HBc antibody	Negative
HCV antibody	Negative
SARS-CoV-2 RT-PCR	Negative
Influenza virus antigen, neuraminidase	Negative
Immunochemistry	
IgG (mg/dL)	1,842
IgA (mg/dL)	334
IgM (mg/dL)	121
IgE (mg/dL)	1,008
Complement activities 50 (U/mL)	<10
Complement 3 (mg/dL)	87
Complement 4 (mg/dL)	2
Rheumatoid factor measurement	8
Antinuclear antibody (ANA)	<40
MPO-ANCA (U/mL)	<1.0
PR-3-ANCA (U/mL)	<1.0
IgG4 (mg/dL)	11.5

**Figure 3:**
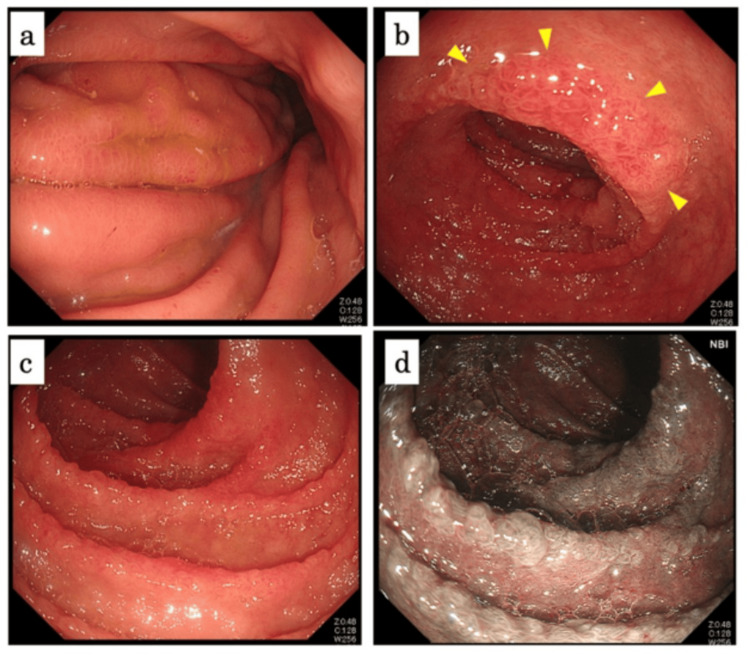
Esophagogastroduodenoscopy on day 22 after admission (a) The stomach. (b-d) The descending part of the duodenum. There was mucosal erythema in the stomach. There were small islands of the remaining villi in the duodenum (b, yellow arrowheads). Those islands were more clearly observed with narrow band imaging (d) than with white light (c).

On day 22 after admission, we examined her with esophagogastroduodenoscopy (EGD), which showed gastric mucosal erythema and duodenal lesions including mucosal erythema and multiple irregular ulcers. Moreover, small islands of the remaining villi were observed in the descending part of the duodenum (Figure 3). These findings suggested that the ulcerative colitis was the unlikely cause of her enterocolitis. Although no differential diagnosis had been established at the time of endoscopy, we found former case reports of IgAV-associated enterocolitis similar to her endoscopic findings in a single day after EGD [3,4].

Pathological findings

**Figure 4:**
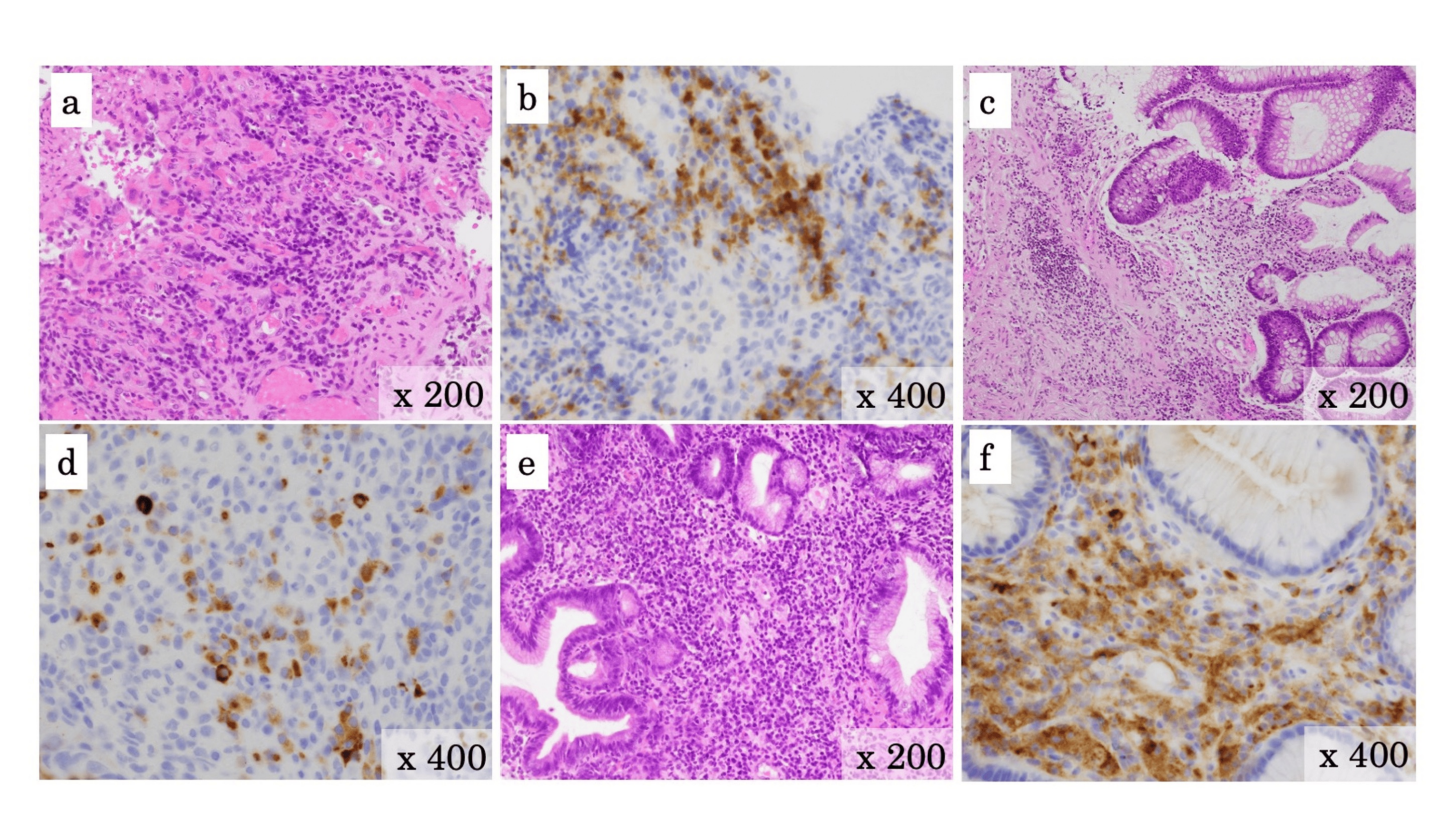
Specimens of mucosal biopsy with hematoxylin and eosin stain (a, b) Specimens of the terminal ileum. (c, d) Specimens of the colon. (e, f) Specimens of the duodenum. Photomicrograph of biopsy from the ileal, colonic, and duodenal lesions showed inflammatory cell infiltrated submucosa, red blood cells leaked around vessels, and leukocytoclastic vasculitis with nuclear dusts (a, c, e). There were IgA-positive lymphocytes gathering around the blood vessels in the submucosa of the ileum and duodenum (b, d, f).

Photomicrograph of biopsy from the duodenal and colonic lesions showed inflammatory cell infiltrated submucosa, red blood cells leaked around vessels, and leukocytoclastic vasculitis with nuclear dusts. Further investigation of mucosal biopsies showed IgA-positive lymphocytes gathering around the blood vessels in the submucosa of the duodenum, ileum, and colon (Figure 4). However, the biopsies did not show granular IgA deposition in the wall of submucosal blood vessels, which is the pathological feature of IgAV-associated enterocolitis. Furthermore, the biopsies did not show granuloma, basal plasma cytosis, or crypt abscess, which are pathological features of Crohn's disease. Therefore, we diagnosed her as a case of undetermined enterocolitis similar to autoimmune vasculitis.

Treatment

**Figure 5:**
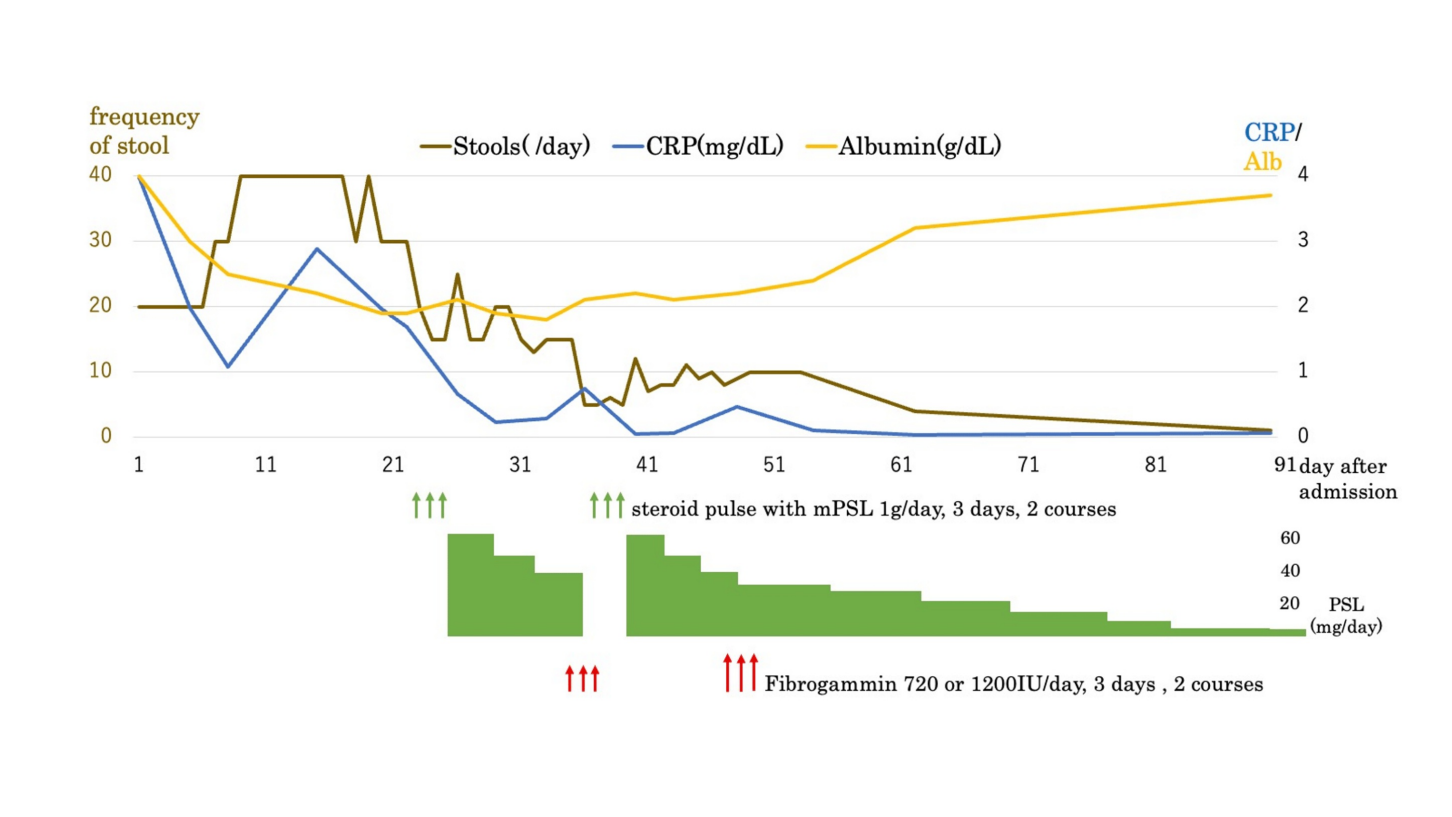
Clinical course after hospitalization

**Figure 6:**
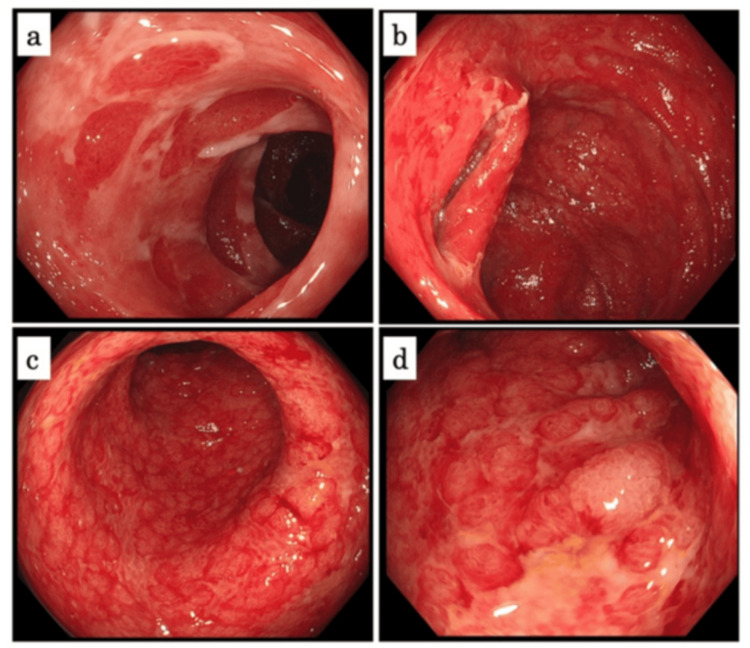
Endoscopic findings of total colonoscopy on day 35 after admission (a) Terminal ileum. (b) The cecum. (c. d) The colon. Findings showed that the area of the ulcer had reduced, and some areas of the colorectum were healed moderately.

**Figure 7:**
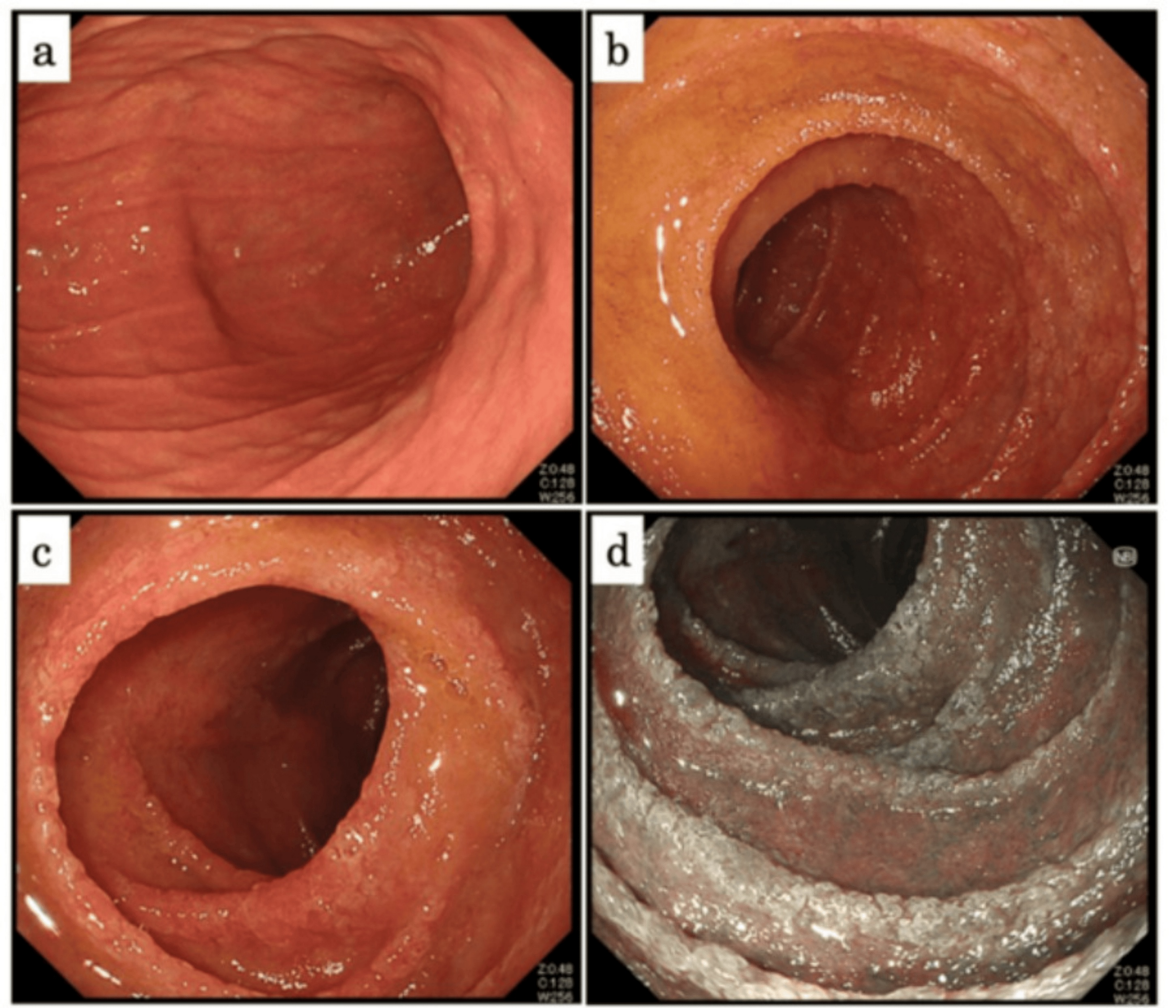
Endoscopic findings of esophagogastroduodenoscopy on day 49 after admission (a) The Stomach. (b-d) The duodenum. Findings demonstrated the disappearance of erythema and regeneration of the duodenal villi.

We considered refractory vasculitis-associated enterocolitis. The patient was started on glucocorticoid therapy on day 23 after admission (Figure 5). Although CRP levels decreased after steroid pulse (methylprednisolone 1g/day for three days), her abdominal symptom did not change, and her diarrhea was still severe. The coagulation factor XIII activity was confirmed as low (41%); therefore, we administered purified coagulation factor XIII concentrate (Fibrogammin P®; CSL Behring, Tokyo, Japan; 720 IU/day for three days) on day 34 after admission. Endoscopic findings of follow-up TCS on day 35 after admission showed that the area of ulcer was reduced and that some areas of the colorectum were healed moderately (Figure 6). We considered her enterocolitis as partially healed. Although laboratory evaluation showed a decreasing trend in CRP levels, serum albumin level was still low (2.1 g/dL on day 36 after admission) and so was coagulation factor XIII activity (63% on day 43 after admission). She still suffered from diarrhea, with around 10 episodes per day. We considered her hitherto therapy insufficient, and thus she was given another steroid pulse therapy and administration of Fibrogammin (1,200 IU/day for three days). Afterward, her diarrhea resolved week by week. The endoscopic findings of EGD on day 49 after admission demonstrated the disappearance of erythema and regeneration of duodenal villi (Figure 7), and she was discharged on day 55 after admission.

After discharge

After tapering off the glucocorticoid, there was no recurrence of symptoms of enterocolitis (Figure 5). The laboratory findings showed the recovery of serum albumin, sodium, and chloride to normal levels (Table 1).

## Discussion

IgAV is a systemic small vessel vasculitis characterized by pathohistological findings of leukocytoclastic vasculitis and IgA-immune deposits in vessel walls [6]. A variety of infections and chemical factors are recognized to be related to IgAV, and many cases have been reported after vaccination. However, the etiology of IgAV remains elusive.

A couple of classification criteria for IgAV has been proposed, which include the manifestation of palpable purpura and not thrombocytopenia for diagnostic purpose [6]. However, some cases of IgAV, especially in adult or elderly patients, reported gastrointestinal symptoms without purpura. Saulsbury reported that abdominal pain preceded the onset of the purpura by 1-14 days in 19% pediatric patients [7]. Symptoms other than purpura are not specific. Therefore, it is difficult to diagnose IgAV without purpura.

Gastrointestinal symptoms, including nausea, vomiting, abdominal pain, and transient paralytic ileus, occur in approximately one-half of adult patients with IgAV, similarly to children [7,8]. Although these symptoms are not specific, distinctive endoscopic findings were reported previously. Esaki et al. reported that the duodenum and small intestine were most frequently involved in patients with IgAV [2]. Kawasaki et al. reported that gastric mucosal erythema and duodenal involvement, including erythema, ulcer, and hematoma-like protrusion, were frequently observed in EGD in patients with IgAV [9]. In the present case, we observed erythema and erosion in the continuous region rather than the skipped region. Enterocolitis in the continuous region is likely observed in the case of bacterial colitis, ischemic colitis, and ulcerative colitis. However, hematoma-like protrusion was observed in TCS, and the findings of EGD were similar to those of TCS. These suggested the differential diagnosis of vasculitis.

The vast majority of patients with IgAV recover spontaneously. However, significant symptoms, such as severe arthralgia, gastrointestinal bleeding, and/or kidney insufficiency, should be managed with systemic administration of glucocorticoid or immunosuppressants (e.g., cyclophosphamide). The efficacy of coagulation factor XIII administration has been reported in patients with IgAV [3,10-12]. The coagulation factor XIII is a proenzyme that promotes fibrin stabilization by forming multiple covalent bonds between fibrin monomers to protect newly formed fibrin from fibrinolysis via cross-linking the α2-plasmin inhibitor [13,14]. Although a definite diagnosis could not be established, a decrease in coagulation factor XIII activity without associated changes in other coagulation factors is distinctive of IgAV [10]. Moreover, the decrease in coagulation factor XIII activity is related to the severity of IgAV. In cases suspected of IgAV, the coagulation factor XIII activity should be measured, and the administration of purified coagulation factor XIII concentrate may be efficient if its activity is decreased.

## Conclusions

We described the case of a patient who experienced suspected IgAV-associated enterocolitis, which was relieved by glucocorticoid therapy and Fibrogammin. As a matter of course, treatment with glucocorticoid therapy and Fibrogammin should be administered for definitively diagnosed IgAV. It is important to considere the differential diagnosis of IgAV in patients with severe erosive duodenitis or small intestinal inflammation, even without purpura. The measurement of coagulation factor XIII activity should be performed in IgAV. In patients in whom IgAV is accompanied by severe enterocolitis, Fibrogammin may be an effective treatment option.
